# Cardiovascular Safety of Anagrelide Hydrochloride versus Hydroxyurea in Essential Thrombocythaemia

**DOI:** 10.1007/s12012-020-09615-0

**Published:** 2020-10-29

**Authors:** Mirjana Gotic, Miklos Egyed, Liana Gercheva, Krzysztof Warzocha, Hans Michael Kvasnicka, Heinrich Achenbach, Jingyang Wu

**Affiliations:** 1 Clinic for Hematology Clinical Centre of Serbia Belgrade, Medical Faculty, University of Belgrade, Koste Todorovica 2, 11000 Belgrade, Serbia; 2 Somogy Megyei Kaposi Mór Oktató Kórház, Kaposvár, 7400 Hungary; 3 Clinic of Hematology, University Hospital St. Marina, 9010 Varna, Bulgaria; 4 Institute of Hematology and Transfusion Medicine, Department of Haematology, 00-791 Warsaw, Poland; 5 Institute of Pathology, University Clinic Wuppertal, University of Witten / Herdecke, Wuppertal, Germany; 6 Research & Development, Shire International GmbH (a Member of the Takeda Group of Companies), 6300 Zug, Switzerland; 7 Research & Development, Shire (a Member of the Takeda Group of Companies), Lexington, MA 02421 USA

**Keywords:** Cardiac safety, Anagrelide, Essential thrombocythaemia, Hydroxyurea, Platelet counts

## Abstract

Essential thrombocythaemia (ET) is a rare myeloproliferative neoplasm. This multicentre, Phase 3b, randomised, open-label, non-inferiority study investigated the cardiac safety, efficacy and tolerability of first-line treatment with anagrelide or hydroxyurea in high-risk ET patients for up to 3 years. Eligible patients aged ≥ 18 years with a diagnosis of high-risk ET confirmed by bone marrow biopsy within 6 months of randomisation received anagrelide (*n* = 75) or hydroxyurea (*n* = 74), administered twice daily. Treatment dose for either compound was titrated to the lowest dose needed to achieve a response**.** Planned primary outcome measures were change in left ventricular ejection fraction from baseline over time and platelet count at Month 6. Planned secondary outcome measures were platelet count change from baseline at Months 3 and 36; percentage of patients with complete or partial response; time to complete or partial response; number of patients with thrombohaemorrhagic events; and changes in white blood cell count or red blood cell count over time. Neither treatment altered cardiac function. There were no significant differences in adverse events between treatment groups, and no reports of malignant transformation. The incidence of disease-related thrombotic or haemorrhagic events was numerically higher in anagrelide-treated patients. Both treatments controlled platelet counts at 6 months, with the majority of patients experiencing complete or partial responses. In conclusion, these results suggest that long-term treatment with anagrelide is not associated with adverse effects on cardiac function. This is one of the few studies using left ventricular ejection fraction assessment and central biopsy reading to confirm the diagnosis of ET.

**Trial registration number**: Clinicaltrials.gov NCT00202644

**Electronic supplementary material:**

The online version of this article (10.1007/s12012-020-09615-0) contains supplementary material, which is available to authorised users.

## Introduction

Essential thrombocythaemia (ET) is a rare myeloproliferative neoplasm characterised by elevated platelet counts, megakaryocyte hyperplasia and enlargement, and one of the following: *JAK2, CALR* or *MPL* mutations, a clonal marker, or lack of evidence for reactive thrombocytosis [1, 2]. In the European Union, incidence of ET ranges from 0.38 to 1.7 per 100,000 per year [3], with patients aged ≥ 60 years or with history of thrombosis at elevated risk of poor outcomes [2]. Long-term prognosis in patients with ET is good, with median survival times of > 30 years in those diagnosed before the age of 60 [4]. However, survival remains worse versus the general population [4], with excess morbidity and mortality related to thrombohaemorrhagic complications [2, 5]. Patients with ET are at risk of thrombosis and, less frequently, haemorrhage of the coronary, cerebral and peripheral vasculatures [6]; many patients experience at least one thrombotic or haemorrhagic event at some point during the course of their disease [7].

For patients with low-risk ET, treatment guidelines recommend observation or low-dose aspirin [8–10]. First-line treatment for high-risk ET includes low-dose aspirin plus cytoreductive therapy; given the evidence for reduced thrombotic complications with platelet-lowering agents, the optimal choice of treatment between hydroxyurea, anagrelide (Xagrid^®^) and interferon-α is unclear [2]. In 2015, the European Society for Medical Oncology guidelines recommended hydroxyurea first line, with anagrelide primarily a second-line therapy option, in line with the European-approved indication for anagrelide [2, 11]. In 2018, European LeukemiaNet also recommended anagrelide as a second-line therapy after hydroxyurea [8].

Anagrelide reduces megakaryocyte hyperproliferation and differentiation, and inhibits cyclic adenosine monophosphate phosphodiesterase 3 (PDE3) and phospholipase A_2_ [11, 12]. In two randomised clinical trials in high-risk ET, anagrelide provided long-term platelet control similar to that with hydroxyurea [13, 14]. PDE3 inhibitors are indicated for the treatment of acute heart failure [15, 16], but cases of cardiomegaly and congestive heart failure have been reported in clinical studies with anagrelide [11], raising potential concerns regarding cardiovascular safety.

### Aims

This study aimed to characterise cardiac safety, efficacy and tolerability of first-line therapy with anagrelide or hydroxyurea in short- and long-term treatment of high-risk ET. Given the positive inotropic and chronotropic effects of PDE3 inhibition, we focused on investigating cardiovascular safety [11, 17].

## Materials and Methods

### Study Design and Patients

This randomised, open-label, Phase 3b study was conducted at 29 sites across Belgium, Bulgaria, France, Hungary, Ireland, Poland, Portugal, Serbia, Slovenia and Spain in high-risk ET patients in accordance with the Declaration of Helsinki and applicable local ethical and legal requirements. Patients provided written informed consent prior to undertaking any study-specific procedures. The trial was registered with ClinicalTrials.gov (NCT00202644).

Eligible patients were aged ≥ 18 years with a diagnosis of ET defined as: (i) platelet count ≥ 600 × 10^9^/L for ≥ 8 weeks; (ii) packed cell volume (PCV) < 0.51 for males or < 0.48 for females, or normal red cell mass in those with a high normal PCV and splenomegaly; and (iii) stainable iron in marrow, normal serum ferritin, or normal red cell mean corpuscular volume. Patients were also required to meet one of the following criteria for high-risk ET: (i) platelet count ≥ 1000 × 10^9^/L; (ii) age ≥ 60 years; or (iii) history of thrombohaemorrhagic events. The genetic markers *JAK2* and *CALR* were not established and routinely used at the initiation of the study and were not indicated as inclusion criteria. Additional inclusion criteria can be found in the Online Resource.

Bone marrow biopsies were performed at screening or within 6 months prior to randomisation to confirm ET diagnosis and were reviewed at the site and centrally using World Health Organization (WHO) criteria [18] by a panel of three haematopathologists reader blinded for the therapy group. Patients with unconfirmed diagnosis of ET by central reading were classified as major protocol deviators but were not excluded from the statistical analysis.

Patients with suspected heart disease, left ventricular ejection fraction (LVEF) < 55%, history of life-threatening malignancy or neoplasia (unrelated to thrombocythaemia), with moderate-to-severe renal impairment (creatinine clearance < 50 mL/min) or with moderate-to-severe hepatic impairment (elevated transaminase levels > 5 times upper limit of normal) were excluded. Additional exclusion criteria can be found in the Online Resource.

### Randomisation and Treatment

Eligible patients were randomised 1:1 to receive either anagrelide hydrochloride or hydroxyurea. Randomisation was performed centrally, with patients allocated randomisation numbers in balanced blocks using an interactive voice response system, and was stratified by age and the presence/absence of prior thrombosis or haemorrhage.

The anagrelide arm initially received 1 mg/day orally in two divided doses (0.5 mg/dose) for at least 1 week, followed by titration to the lowest effective dose to achieve a response. A maximum dosing increment of 0.5 mg/day in any one week was permitted; the maximum single dose was 2.5 mg, with total daily dose limited to 10 mg. Patients randomised to hydroxyurea began at 1000 mg/day orally in two divided doses (500 mg/dose), followed by titration to the lowest effective dose to achieve a response. The anagrelide dose was in line with the approved dosing schedule outlined in the Summary of Product Characteristics [11]. Dose reduction of either agent was allowed if adverse events (AEs) were not tolerable. During an initial, 6-month titration period, patients were required to visit the clinic at least every 2 weeks until an acceptable platelet count was achieved (verified over two consecutive visits at least 4 weeks apart).

### Outcomes and Assessments

The primary objective was to compare cardiovascular function and safety of short- and long-term anagrelide or hydroxyurea use, as assessed by echocardiography. The primary endpoint was LVEF (primary outcome analysis). The primary efficacy outcome was platelet counts at 6 months. Secondary outcome measures were platelet counts at 3 and 36 months, complete response (platelet count < 400 × 10^9^/L; CR) or partial response (platelet count 400–600 × 10^9^/L with a reduction of ≥ 200 × 10^9^/L; PR) rates (confirmed over two consecutive visits ≥ 1 month apart), average time to response, incidence of disease-related thrombohaemorrhagic events, and cytoreductive impact on white and red blood cell lines.

Patients attended mandatory monthly clinic visits for the first 6 months, followed by quarterly visits up to Month 12, then visits every 6 months for the remaining 2 years. Patients were followed for up to 3 years. At each visit, patients underwent assessment of vital signs, biochemistry, haematology, urinalysis and AEs. In patients reporting pre-defined cardiovascular symptoms, 24-h Holter monitoring was performed and evaluated centrally according to an agreed protocol. Physical examination, 12-lead electrocardiogram and echocardiogram assessments were performed at screening, Months 1, 2, 3 and 6 of the titration period, and all subsequent scheduled visits. Resting LVEF was assessed with a protocol that specified equipment, site certification and conduct of the procedure. Recordings were evaluated by blinded review at a central reading centre. The central LVEF reading was used for evaluation.

### Statistical Analysis

Analyses were performed using SAS^®^ version 9.3 (SAS Institute, Cary, NC, USA), with comparisons between groups made using 95% confidence intervals (CIs), where appropriate. Three analysis populations were defined: i) safety population (patients who received ≥ 1 dose of study medication); ii) full analysis set (FAS, patients who received ≥ 1 dose of study medication and had a pre-treatment and ≥ 1 post-baseline LVEF measurement); and 3) per-protocol (PP) population (FAS subjects with no major protocol deviations).

For primary outcome analysis, LVEF data were analysed using a mixed-effects model, including treatment, age category and presence of previous thrombosis or haemorrhage as fixed effects, with subject and time of measurement as random effects, and platelet count at baseline as a covariate. Linear regression was used to obtain the response (slope) and intercept in LVEF. Least-square (LS) mean values for each treatment group, the difference between groups and two-sided 95% CIs for the difference between groups were calculated. If the 95% CI for the difference in LVEF slopes between treatment groups lied entirely above − 2%/year, the effect of anagrelide on LVEF was considered non-inferior to hydroxyurea (assuming this condition was met for both FAS and PP populations).

Mean platelet count at Month 6 was analysed using analysis of covariance (ANCOVA), with treatment, age category and previous thrombosis or haemorrhage as main effects, and platelet count at baseline as a covariate. LS means, treatment differences and 95% confidence intervals (CIs) were calculated as above. If the lower limit of the 95% CI for the difference between treatments (hydroxyurea minus anagrelide) in the FAS population was greater than − 100 × 10^9^/L, the effect of anagrelide on platelet count was considered non-inferior to hydroxyurea. If Month 6 data were missing, last observation carried forward [LOCF] imputation was used.

Sample size was determined based on the anticipated slope in LVEF over time. Assuming a measurement error standard deviation (SD) of 10% and a between-subject SD in the slopes of 3%/year, the SD of the estimates of the slopes was 4.26%, given the planned assessment schedule for this 3-year study. A sample size of 73 patients per treatment group provided 80% probability that the 95% CI for the difference between anagrelide and hydroxyurea fell entirely above the lower limit of the non-inferiority interval of − 2%/year. Allowing for an assumed unevaluable rate of 20%, we planned to randomise 92 patients per treatment group.

## Results

### Study Population

**Fig. 1:**
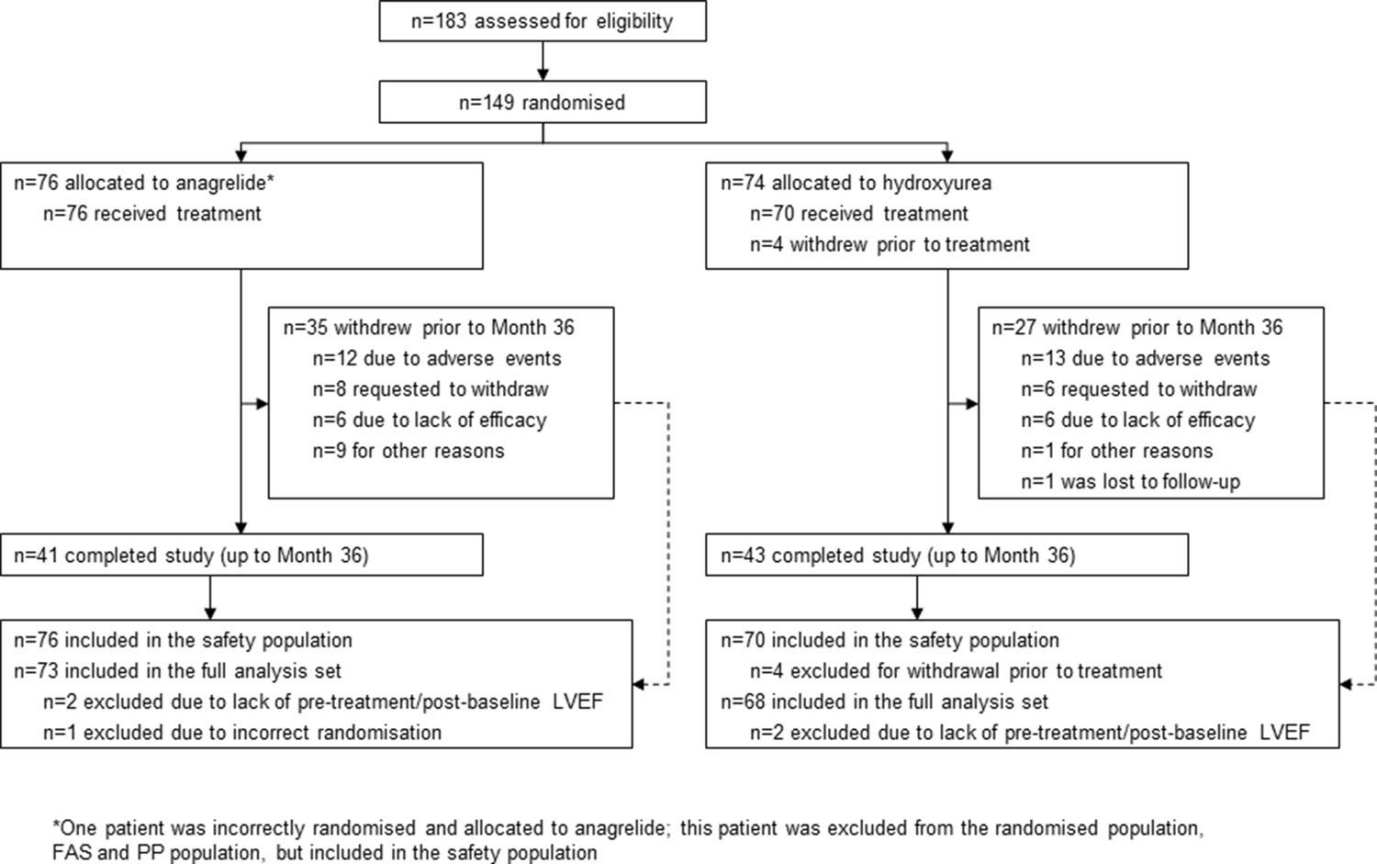
Patient disposition. *LVEF*, left ventricular ejection fraction; *FAS*, full analysis set; *PP*, per protocol

The study was conducted between 13 January 2006 and 15 December 2015. Overall, 183 patients were screened, with 149 patients randomised (Fig. 1). One additional patient was incorrectly randomised to anagrelide and was excluded from the FAS and PP populations but included in the safety population. In the first 6 months of the study, 25 patients withdrew across both arms, predominantly due to AEs (*n* = 10) and patient requests (*n* = 7). From Month 6 to Month 36, an additional 37 patients withdrew across both arms, primarily due to AEs (*n* = 15), patient requests (*n* = 7) and lack of efficacy (*n* = 11). The overall withdrawal rate up to Month 36 was 46.1% with anagrelide and 38.6% with hydroxyurea.

Baseline demographics were balanced between treatment groups (Table 1). Most patients were female (69.2%) and Caucasian (98.6%), and the mean age was 52.5 years. The incidence of patients aged ≥ 60 years was comparable between treatment groups: 46.1% anagrelide patients vs. 45.7% hydroxyurea patients. Mean time since diagnosis was lower in the anagrelide (8.7 months) than the hydroxyurea group (14.6 months). Fewer patients had platelet count ≥ 1000 × 10^9^/L in the anagrelide group (56.6%) than in the hydroxyurea group (71.4%). A similar proportion of patients fulfilled one risk category in the two groups (82.9% for anagrelide and 71.4% for hydroxyurea); however, fewer patients in the anagrelide group fulfilled two risk categories compared with the hydroxyurea group (15.8% and 27.1%, respectively).

**Table 1:** Patient demographics and characteristics (safety population)

Parameter	Anagrelide (*n* = 76)	Hydroxyurea (*n* = 70)	Total (*n* = 146)
Age (years)			
Mean (SD)	52.1 (16.10)	52.9 (15.80)	52.5 (15.91)
Median (range)	53.0 (18–78)	57.0 (22–84)	55.0 (18–84)
Age category (years), *n* (%)			
18– < 40	20 (26.3)	16 (22.9)	36 (24.7)
40– < 60	21 (27.6)	23 (32.9)	44 (30.1)
60– < 75	31 (40.8)	28 (40.0)	59 (40.4)
≥ 75	4 (5.3)	3 (4.3)	7 (4.8)
Gender, *n* (%)			
Female	56 (73.7)	45 (64.3)	101 (69.2)
Race, *n* (%)			
Caucasian	75 (98.7)	69 (98.6)	144 (98.6)
Time since ET diagnosis (months)			
Mean (SD)	8.7 (13.65)	14.6 (28.29)	11.5 (22.04)
High-risk category fulfilled, *n* (%)			
Platelet count ≥ 1000 × 10^9^/L	43 (56.6)	50 (71.4)	93 (63.7)
Age ≥ 60 years	35 (46.1)	32 (45.7)	67 (45.9)
Previous history of thrombotic or haemorrhagic events	12 (15.8)	9 (12.9)	21 (14.4)
Number of high-risk categories fulfilled, *n* (%)			
1	63 (82.9)	50 (71.4)	113 (77.4)
2	12 (15.8)	19 (27.1)	31 (21.2)
3	1 (1.3)	1 (1.4)	2 (1.4)
Bone marrow biopsy result—study centre result, *n* (%)			
ET	72 (94.7)	68 (97.1)	140 (95.9)
MPN, unclassifiable	4 (5.3)	2 (2.9)	6 (4.1)
Bone marrow biopsy result (WHO classification)—central reading / final diagnosis, *n *(%)			
ET	41 (53.9)	52 (74.3)	93 (63.7)
Pre-PMF (fibrosis grade 0 or 1)	10 (13.2)	5 (7.1)	15 (10.3)
Overt PMF (fibrosis grade 2)	5 (6.6)	4 (5.7)	9 (6.2)
Polycythaemia vera	3 (3.9)	2 (2.9)	5 (3.4)
MPN, unclassifiable	5 (6.6)	2 (2.9)	7 (4.8)
Limited material/non-representative biopsy	12 (157)	5 (7.1)	17 (11.6)
Prior use of platelet aggregation inhibitors, *n* (%)	30 (39.5)	22 (31.4)	52 (35.6)

*ET* essential thrombocythaemia; *MPN* myeloproliferative neoplasm; *PMF* primary myelofibrosis; *SD* standard deviation; *WHO* World Health Organization

Bone marrow ET diagnosis done locally was confirmed by central review using WHO criteria in 63.7% of patients (53.9% vs. 74.3% in the anagrelide and hydroxyurea groups, respectively). Fifteen anagrelide-treated patients (19.7%) and nine hydroxyurea-treated patients (12.9%) had a confirmed diagnosis of primary myelofibrosis (fibrosis grade 0–2); three anagrelide-treated patients (3.9%) and two hydroxyurea-treated patients (2.9%) had a confirmed diagnosis of polycythaemia vera (Table 1).

Prior medication was used in 32 patients (42.1%) in the anagrelide group and 25 patients (35.7%) in the hydroxyurea group. Platelet aggregation inhibitors, excluding heparin, were used in 30 anagrelide-treated patients (39.5%) and 22 hydroxyurea-treated patients (35.7%). During the study, 50 anagrelide-treated patients (65.8%) and 43 hydroxyurea-treated patients (61.4%) used concomitant medications, the most frequent being paracetamol, augmentin and amoxicillin.

The main reasons for exclusion from the PP populations were use of disallowed medications (34.2% of anagrelide-treated patients and 22.1% of hydroxyurea-treated patients, respectively), and diagnosis unconfirmed by bone marrow biopsy.

### Primary Outcome: Safety

The mean (SD) daily doses of anagrelide and hydroxyurea were 1.73 (0.679) mg and 1082.15 (353.8) mg, respectively, with a mean (SD) treatment duration of 696 (444.5) days for anagrelide and 801 (424.6) days for hydroxyurea. There was no statistically significant difference in effect on LVEF between anagrelide and hydroxyurea groups. The LS mean difference in LVEF slope between groups was − 0.34%/year (95% CI − 0.86 to 0.18) for anagrelide versus hydroxyurea in the FAS population, and − 0.93% per year (95% CI − 1.62 to − 0.24) in the PP population. LVEF mean changes from baseline to Month 36 were small in both groups, ranging from a maximum increase of 1.2% to a maximum decrease of 2.0% with anagrelide (mean [SD] LVEF at baseline: 66.4% [4.81] and at Month 36: 64.8% [3.17]), and a maximum decrease of 1.7% with hydroxyurea. No mean increase with hydroxyurea was observed at any time point in the FAS population (mean [SD] LVEF at baseline: 66.9% [4.59] and at Month 36: 65.1% [3.73]) (Table 2). Shifts in LVEF in the PP population were generally comparable to the FAS population. Larger fluctuations in LVEF were observed in both groups with increasing age but not with prior thrombosis or haemorrhage. All individual LVEF values were above the protocol criterion for withdrawal of a subject (< 50%), except for a single anagrelide patient with an LVEF of 48% at Month 6; this patient had LVEF > 50% at all other visits and was not withdrawn. The proportion of patients shifting from a normal to an abnormally low LVEF (< 52% for males; < 54% for females) was low in both groups, with shifts observed in three anagrelide patients and one hydroxyurea patient; all shifts occurred within the first 9 months of treatment (FAS data; PP population results were comparable).

**Table 2:** Left ventricular ejection fraction by visit (FAS population)

	Anagrelide *n* = 73		Hydroxyurea *n* = 68	
	LVEF at visit (%)	Change from baseline^a^ (%)	LVEF at visit (%)	Change from baseline ^a^ (%)
Baseline				
*n*	73		68	
Mean (SD)	66.4 (4.81)		66.9 (4.59)	
Median (min, max)	66.0 (55, 88)		67.0 (55, 78)	
Month 1				
*n*	71	71	64	64
Mean (SD)	66.8 (3.79)	0.5 (4.68)	65.6 (4.07)	− 1.1 (4.73)
Median (min, max)	67.0 (58, 77)	1.0 (− 13, 10)	66.0 (59, 79)	− 1.0 (− 16, 12)
Month 2				
*n*	68	68	63	63
Mean (SD)	67.5 (4.00)	1.2 (5.80)	66.7 (4.66)	0 (5.03)
Median (min, max)	67.0 (56, 80)	1.0 (− 23, 19)	67.0 (58, 81)	0 (− 13, 16)
Month 3				
*n*	67	67	62	62
Mean (SD)	66.4 (4.90)	0.1 (5.31)	66.3 (4.19)	− 0.4 (3.94)
Median (min, max)	66.0 (57, 87)	− 1.0 (− 13, 24)	66.0 (50, 73)	− 1.0 (− 11, 11)
Month 6				
*n*	59	59	60	60
Mean (SD)	65.9 (5.13)	− 0.5 (5.68)	66.1 (3.97)	− 0.6 (3.95)
Median (min, max)	66.0 (48, 80)	0 (− 23, 11)	66.0 (56, 77)	− 0.5 (− 9, 11)
Month 9				
*n*	52	52	52	52
Mean (SD)	65.9 (5.12)	− 0.8 (4.78)	64.8 (3.97)	− 1.5 (5.15)
Median (min, max)	66.0 (51, 77)	0 (− 14, 9)	65.0 (56, 75)	− 2.0 (21, 10)
Month 12				
*n*	45	45	52	52
Mean (SD)	65.9 (3.73)	− 0.8 (6.61)	65.7 (4.12)	− 0.6 (5.67)
Median (min, max)	66.0 (56, 72)	− 1.0 (− 18, 15)	65.0 (58, 78)	0.0 (− 13, 13)
Month 18				
*n*	41	41	48	48
Mean (SD)	64.4 (3.92)	− 2.0 (5.54)	64.7 (4.47)	− 1.2 (4.84)
Median (min, max)	65.0 (54, 73)	− 2.0 (− 15, 8)	65.0 (54, 74)	− 1.0 (− 23, 7)
Month 24				
*n*	40	40	49	49
Mean (SD)	64.7 (3.88)	− 1.8 (5.84)	64.2 (3.72)	− 0.2 (6.17)
Median (min, max)	65.0 (57, 73)	− 1.0 (− 18, 16)	65.0 (54, 72)	0 (− 23, 12)
Month 30				
*n*	40	40	45	45
Mean (SD)	64.7 (3.41)	− 1.8 (5.84)	65.5 (4.08)	− 0.2 (5.38)
Median (min, max)	65.0 (58, 71)	− 1.5 (− 23, 10)	65.0 (55, 74)	0 (− 19, 15)
Month 36				
*n*	40	40	44	44
Mean (SD)	64.8 (3.17)	− 1.7 (6.55)	65.1 (3.73)	− 0.6 (5.46)
Median (min, max)	65.5 (58, 71)	− 1.0 (− 25, 15)	65.0 (57, 75)	− 1.0 (− 14, 11)

*LVEF*, left ventricular ejection fraction; *SD*, standard deviation

^a^Baseline corresponds to the first valid (non-missing interpretation) observation obtained at the baseline visit; if this was missing, the screening value was used

A similar proportion of patients experienced treatment-emergent AEs (TEAEs) in anagrelide (76.3%) and hydroxyurea groups (71.4%) (Table 3). Treatment-related TEAEs were reported in a similar proportion of patients in each group (46.1% and 42.9% in anagrelide and hydroxyurea groups, respectively). Fifty percent of patients in the anagrelide group and 45.7% of patients in the hydroxyurea group experienced their first TEAE during Months 1–3. Serious TEAEs were reported in 17 patients (22.4%) in the anagrelide group and in 13 patients (18.6%) in the hydroxyurea group. Except for three reports of ischaemic stroke in the anagrelide group, all other serious TEAEs were reported once per group. Three patients with serious TEAEs in the anagrelide group died, one each of ischaemic stroke, pulmonary embolism or sudden death.

**Table 3:** Treatment-emergent adverse events occurring in ≥ 5% of patients (safety population)

MedDRA system organ class preferred term	Anagrelide *n* = 76 *n* (%)	Hydroxyurea *n* = 70 *n* (%)
Patients with ≥ 1 TEAE	58 (76.3)	50 (71.4)
Nervous system disorders	29 (38.2)	8 (11.4)
Headache	19 (25.0)	1 (1.4)
Cardiac disorders	23 (30.3)	4 (5.7)
Palpitations	18 (23.7)	0
Infections and infestations	22 (28.9)	18 (25.7)
Urinary tract infection	4 (5.3)	2 (2.9)
Pharyngitis	2 (2.6)	6 (8.6)
Upper respiratory tract infection	4 (5.3)	1 (1.4)
Nasopharyngitis	2 (2.6)	5 (7.1)
General disorders and administration site conditions	20 (26.3)	9 (12.9)
Asthenia	5 (6.6)	4 (5.7)
Chest pain	4 (5.3)	1 (1.4)
Gastrointestinal disorders	18 (23.7)	14 (20.0)
Diarrhoea	6 (7.9)	3 (4.3)
Vascular disorders	14 (18.4)	4 (5.7)
Hypertension	9 (11.8)	1 (1.4)
Musculoskeletal and connective tissue disorders	13 (17.1)	12 (17.1)
Arthralgia	6 (7.9)	2 (2.9)
Respiratory, thoracic and mediastinal disorders	9 (11.8)	7 (10.0)
Epistaxis	4 (5.3)	2 (2.9)
Skin and subcutaneous tissue disorders	8 (10.5)	11 (15.7)
Investigations	7 (9.2)	8 (11.4)
Ear and labyrinth disorders	7 (9.2)	0
Vertigo	5 (6.6)	0
Blood and lymphatic system disorders	6 (7.9)	18 (25.7)
Anaemia	4 (5.3)	8 (11.4)
Leukopenia	1 (1.3)	7 (10.0)
Neutropenia	0	5 (7.1)
Eye disorders	5 (6.6)	2 (2.9)
Metabolism and nutrition disorders	4 (5.3)	1 (1.4)
Injury, poisoning and procedural complications	3 (3.9)	5 (7.1)

*MedDRA* Medical Dictionary for Regulatory Activities; *TEAE* treatment-emergent adverse event

Treatment-related serious TEAEs were reported in 5.3% and 2.9% of patients in the anagrelide and hydroxyurea groups, respectively. TEAEs leading to discontinuation occurred in 18.4% and 18.6% of patients in the anagrelide and hydroxyurea groups, respectively. TEAEs leading to discontinuation were primarily vascular (6.6%), cardiac (6.6%) and nervous system disorders (3.9%) in the anagrelide group, and primarily vascular (4.3%), skin (4.3%) and gastrointestinal disorders (2.9%) in the hydroxyurea group. There were no reports of transformations to myelofibrosis or acute myeloid leukaemia. Overall, three patients (4.3%) in the hydroxyurea group and one patient (1.3%) in the anagrelide group had a TEAE in the neoplasm benign, malignant and unspecified (including cysts and polyps) system organ class, all of which were classed as serious; none were suspected to be treatment related.

Overall, the incidence of disease-related thrombotic or haemorrhagic events in the FAS population was numerically higher with anagrelide (41.1%) versus hydroxyurea (23.5%). In total, seven patients (9.6%) in the anagrelide group and three (4.4%) in the hydroxyurea group experienced major thrombohaemorrhagic events. The difference between groups primarily arose from five thrombotic events in four anagrelide-treated patients; one patient each experienced cerebral infarction and hemiparesis, and three patients experienced ischaemic stroke. Four of the seven anagrelide-treated patients with major thrombohaemorrhagic events had history of thrombosis or haemorrhage; none of the three patients with such events in the hydroxyurea group had a similar history. A *post-hoc* multivariate Cox regression model analysis was performed to identify risk factors for major thrombohaemorrhagic events. The model included gender, age, platelet count, history of thrombohaemorrhagic events based on ET history or medical history, prior cardiovascular events, WHO-defined risk factors and prior anticoagulant/anti-aggregant therapy as covariates. After adjusting for 13 risk factors, rates of major thrombohaemorrhagic events were higher in patients receiving, rather than not receiving, concomitant anti-coagulants (*p* = 0.0002). Rates were also higher in patients with two or three WHO-specified risk factors compared with patients having only one risk factor (*p* = 0.0020).

Mean leukocyte count was mildly reduced from baseline to Month 36 in the anagrelide group (from 9.13 to 7.86 × 10^9^/L), with a larger decrease observed in the hydroxyurea group (from 10.24 to 6.01 × 10^9^/L). Mean red blood cell count remained moderately stable from baseline to Month 36 in the anagrelide group (4.76 and 4.31 × 10^12^/L, respectively), with a larger decrease in the hydroxyurea group (4.79 and 3.46 × 10^12^/L, respectively).

### Efficacy Outcomes

At Month 6, platelet count was adequately controlled in most patients in both groups; median platelet count was 398.5 × 10^9^/L with anagrelide and 389.5 × 10^9^/L with hydroxyurea in the FAS population (Table 4), with comparable counts in the PP population (365.0 × 10^9^/L and 389.0 × 10^9^/L with anagrelide and hydroxyurea, respectively).

**Table 4:** Platelet count at Months 3, 6 and 36 (FAS population)

Study visit	Anagrelide *n* = 73		Hydroxyurea *n* = 68	
	Observed value (× 10^9^/L)	Change from baseline^a^	Observed value (× 10^9^/L)	Change from baseline^a^
Screening				
*n*^b^	73	NA	68	NA
Mean (SD)	1056.5 (330.03)	NA	1177.6 (459.50)	NA
Median (min, max)	1016.0 (552, 2882)	NA	1094.0 (621, 3772)	NA
Baseline				
*n*^b^	73	NA	68	NA
Mean (SD)	1076.0 (294.42)	NA	1159.1 (435.30)	NA
Median (min, max)	1027.0 (601, 2077)	NA	1116.5 (583, 3432)	NA
Month 3				
*n*^b^	67	67	62	62
Mean (SD)	487.5 (211.44)	− 597.3 (331.70)	396.4 (170.33)	− 745.6 (345.46)
Median (min, max)	435.0 (160, 1314)	− 626.0 (− 1642, 121)	372.5 (82, 1219)	− 655.0 (− 2326, − 214)
Month 6				
*n*^b^	60	60	58	58
Mean (SD)	418.6 (135.96)	− 660.4 (278.22)	396.0 (144.07)	− 751.6 (294.26)
Median (min, max)	398.5 (181, 779)	− 657.5 (− 1301, 134)	389.5 (102, 833)	− 729.0 (− 2037, − 264)
Month 36				
*n*^b^	40	40	43	43
Mean (SD)	384.9 (126.29)	− 742.2 (262.14)	446.6 (144.35)	− 727.6 (349.81)
Median (min, max)	386.0 (193, 748)	− 741.5 (− 1297, − 223)	435.0 (170, 886)	− 596.0 (− 1924, − 201)

*NA* not applicable; *SD* standard deviation

^a^Baseline corresponds to the first valid (non-missing interpretation) observation obtained at the baseline visit; if this was missing, the screening value was used

^b^If a value was missing at a visit, the subject was not included in the n for that visit

In the FAS population, LS means at Month 6 were lower with hydroxyurea than with anagrelide (421.9 × 10^9^/L and 522.3 × 10^9^/L, respectively). The LS mean difference between hydroxyurea and anagrelide at Month 6 was –100.5 × 10^9^/L (95% CI − 179.42 to − 21.49), indicating an inferior effect of anagrelide on platelet count. In contrast, in the PP population, LS mean difference between hydroxyurea and anagrelide at the same time point was 23.0 × 10^9^/L (95% CI − 63.00 to 109.04), indicating a non-inferior effect of anagrelide on platelet count (PP population LS mean platelet count: 408.2 × 10^9^/L for hydroxyurea; 385.2 × 10^9^/L for anagrelide). A subsequent *post-hoc* analysis of the FAS population (observed) revealed that LS mean platelet counts at 6 months were 393.2 × 10^9^/L and 425.9 × 10^9^/L with hydroxyurea and anagrelide, respectively. In this analysis, LS mean difference was − 32.7 × 10^9^/L (95% CI − 83.00 to 17.52), indicating a non-inferior effect of anagrelide on platelet count. A *post-hoc* mixed-effects model analysis was also conducted for the FAS population, including treatment, month, age category and presence of prior thrombohaemorrhagic event as main effects, and baseline platelet count as a covariates. Based on this model, LS mean difference at Month 6 was − 45.5 × 10^9^/L (95% Cl − 96.66 to 5.71), again indicating a non-inferior effect of anagrelide on platelet count (LS mean platelet count: 396.8 × 10^9^/L for hydroxyurea; 442.2 × 10^9^/L for anagrelide).

Changes in mean platelet counts from baseline at Months 3 and 36 indicated an early onset of response and sustained response in both treatment groups (Table 4), with a greater effect of hydroxyurea on platelet count prior to Month 6 and comparable effects thereafter (Online Resources 1a-c). In the FAS population, a similar proportion of patients had CR with anagrelide (58.9%) or hydroxyurea (58.8%); an additional 21.9% and 27.9% of patients, respectively, experienced PR. In the PP population, a numerically higher proportion of patients in the anagrelide group (77.3%) had CR compared with the hydroxyurea group (57.9%), with a further 18.2% and 31.6% of patients, respectively, experiencing PR. Times to CR or PR were longer with anagrelide than with hydroxyurea for both FAS and PP populations, reflecting the initially greater effect of hydroxyurea on platelet count described earlier. Median time to CR and CR/PR in the FAS population was 177.0 and 61.0 days, respectively, for anagrelide and 123.0 and 47.0 days, respectively, for hydroxyurea. In the PP population, median time to CR and CR/PR for anagrelide was 147.0 and 30.0 days, respectively, and 134.0 and 55.0 days, respectively, for hydroxyurea.

## Discussion

This study suggests that first-line long-term treatment with anagrelide is not associated with significant changes in cardiac function in patients with high-risk ET. Changes from baseline in LVEF were small at each visit with anagrelide or hydroxyurea, with few patients in either group shifting from normal to abnormal values. Given concerns that PDE3 inhibition with anagrelide and resulting positive inotropic and chronotropic effects could lead to adverse changes in cardiac function, the lack of significant impact on LVEF with long-term use in this study is an important observation, enhancing understanding of the safety profile of anagrelide. These results support data from previous observational and retrospective studies showing a low incidence of cardiovascular-related AEs necessitating discontinuation in patients treated with anagrelide [19, 20].

In line with previous studies [13, 14], anagrelide and hydroxyurea both provided adequate platelet control in most patients, with similar proportions of patients experiencing CR or PR. Platelet counts indicated an early onset of response with both agents, with hydroxyurea appearing to have an initially stronger effect, with shorter median times to CR or PR compared with anagrelide. However, by Month 6, platelet counts were comparable between treatments, and responses were sustained with either agent until the end of the 3-year study. Although FAS population ANCOVA analysis results indicated that reductions in platelet count at Month 6 were inferior with anagrelide versus hydroxyurea, non-inferiority was seen in the PP population and in subsequent *post-hoc* sensitivity analyses of the FAS dataset.

Thrombohaemorrhagic events are leading causes of poor outcomes in ET patients; a key therapy goal is reducing the incidence of such events [5, 8]. Previous studies have provided inconsistent data on the relative effects of anagrelide and hydroxyurea on thrombohaemorrhagic events. The UK-PT1 study suggested increased incidence of arterial thrombosis and serious haemorrhage, and decreased risk of venous thromboembolism for anagrelide in combination with aspirin versus hydroxyurea in newly and previously treated high-risk ET patients [14]. In contrast, the ANAHYDRET study enrolled treatment-naïve, high-risk ET patients, diagnosed according to WHO criteria and demonstrated no difference in thrombohaemorrhagic events between anagrelide and hydroxyurea, with use of concomitant aspirin restricted to select patients [13]. However, the ANAHYDRET study was a non-inferiority study, so may not have been powered to detect between-group differences. Our study design has parallels with ANAHYDRET, as it included assessment of thrombohaemorrhagic events as a secondary endpoint but lacked sufficient power to detect differences between treatments on this endpoint. Overall, the incidence of thrombohaemorrhagic events was numerically higher with anagrelide than hydroxyurea, with differences in major event rates primarily due to five thrombotic events in the nervous system disorder class in four anagrelide patients, two of whom had a history of thrombohaemorrhagic events. Incidence of haemorrhagic events was not increased with anagrelide versus hydroxyurea. Recent data from 280 ET patients in a myeloproliferative neoplasms patient registry followed for a median of 6 years provide further long-term perspectives—similar incidences of major arterial and venous thrombotic events were reported with anagrelide treatment versus hydroxyurea plus aspirin treatment, but significantly fewer minor events were reported with anagrelide (*p* < 0.001) [21]. *Post-hoc* exploratory analyses of our patient cohort identified numerically higher rates of thrombohaemorrhagic events in patients receiving concomitant anti-coagulatory therapy, supporting the current product label.

Our study has similarities to a prospective observational study by Tortorella et al., which assessed cardiac safety of anagrelide in 38 patients with ET aged 19–67 [20]. Similarly, we chose a patient population of ≥ 18 years, as the age range of patients affected by ET is wide, with a small number of young patients affected [22]. However, the focus of our study population was high-risk patients with ET. Both studies evaluated LVEF. Tortorella et al. results showed that CV AEs were easily managed, with low withdrawal rates due to CV AEs [20]. A retrospective registry study by Gugliotta et al. also assessed CV AEs during anagrelide treatment, and found that most CV AEs were mild and easily manageable [19]. In order to advance these results and assess cardiac safety in patients with ET on a larger scale, our study enrolled a larger patient population in a randomised controlled trial, with LVEF as the primary endpoint.

The incidence of TEAEs was comparable between groups, with most patients experiencing their first TEAE early in the study. Overall, anagrelide and hydroxyurea were well tolerated, with similar rates of discontinuations due to TEAEs. Serious TEAEs occurred in more patients treated with anagrelide versus hydroxyurea, primarily because of three ischaemic stroke events in the anagrelide group. Avoiding increased risk of malignant transformation is a vital in selecting cytoreductive therapy [8, 23]; there were no reports of transformation to myelofibrosis or acute myeloid leukaemia in this long-term study.

The sample size enrolled and the relatively high rate of subject withdrawal are the limitations of this study; 25 patients withdrew prior to Month 6 and an additional 37 patients withdrew by Month 36. Clusters of withdrawals were likely related to study site processes rather than to drug-related concerns. Additional challenges were protocol deviations; most patients had ≥ 1 major protocol deviation during the study, which reduced the PP dataset. Deviations were due to use of prohibited medications or to lack of a centrally confirmed ET diagnosis according to WHO criteria. We specifically excluded patients with suspected heart disease or low LVEF at baseline, limiting the applicability of our conclusions to populations without such risk factors. However, our study enrolled patients from approximately 29 sites across Europe, which aids the generalisability of the results, although could also have contributed to the relatively high dropout rate. Another limitation is the rate of misdiagnosis, with ~ 36% of patients receiving a different diagnosis to ET following central review. This highlights the potential difficulties with diagnosing ET, and further reduced the patient pool in this study.

LVEF was chosen as the primary endpoint, as it is considered a sufficiently sensitive measure to evaluate changes in cardiac function [24]. LVEF was measured at pre-defined intervals, and the response (slope) for each patient was obtained using linear regression. A sample size of 73 patients per treatment group was sufficient to give an 80% probability that the 95% CI for the difference between anagrelide and hydroxyurea fell entirely above the lower limit of the non-inferiority interval. An analysis of three independent trials showed that all-cause mortality and cardiovascular death declined with an increasing ejection fraction of 45%, after which the risk of these outcomes remained stable with increasing LVEF [25]. However, we recognise that LVEF may have limitations as a cardiac assessment marker, for example, in heart failure with reduced EF and ischaemic disease, where both systolic and diastolic ventricular volumes may be increased, so although stroke volume is preserved, LVEF is reduced [26]. Despite its limitations, LVEF remains an important marker of cardiovascular risk; future studies may benefit from assessing cardiovascular biomarkers alongside LVEF.

Other possible markers of cardiotoxicity include palpitations (tachyarrhythmias) and chest pain (angina), both of which were more common in patients receiving anagrelide compared with those receiving hydroxyurea. Further investigation into other forms of cardiotoxicity with anagrelide may be warranted. In addition, the observation period of 6 months may have been sufficient to detect differences between the groups in terms of LVEF, but not other forms of cardiotoxicity.

In summary, our study suggests that long-term treatment with anagrelide in high-risk ET patients is not associated with adverse effects on cardiac function. Despite the limitations, this is one of the few studies using LVEF assessment and central biopsy reading to confirm the diagnosis of ET.

## Electronic supplementary material

Below is the link to the electronic supplementary material.

Supplementary file1 (DOCX 40 kb)

## Data Availability

The datasets, including the redacted study protocol, redacted statistical analysis plan and individual participants’ data supporting the results reported in this article, will be made available within three months from initial request, to researchers who provide a methodologically sound proposal. The data will be provided after its de-identification, in compliance with applicable privacy laws, data protection and requirements for consent and anonymisation.
