# Supplementary material for: Cardiovascular Safety of Anagrelide Hydrochloride versus Hydroxyurea in Essential Thrombocythaemia
Source: Cardiovasc Toxicol. 2020 Oct 29;21(3):236–47. doi: 10.1007/s12012-020-09615-0 (PMC7847982; doi:10.1007/s12012-020-09615-0)
Supplement: Supplementary file 1 — Supplementary file1 (DOCX 40 kb) [file 12012_2020_9615_MOESM1_ESM.docx]

**Appendix: Additional inclusion and exclusion criteria**

**A Phase 3b, Randomised, Open-label Study to Compare the Cardiovascular Safety of Anagrelide Hydrochloride versus Hydroxyurea in High-risk Essential Thrombocythaemia Patients**

Mirjana Gotic,^1^ Miklos Egyed,^2^ Liana Gercheva,^3^ Krzysztof Warzocha,^4^ Hans Michael Kvasnicka,^5^ Heinrich Achenbach,^6^ Jingyang Wu^7^

^1^ Clinic for Hematology Clinical Centre of Serbia Belgrade, Medical Faculty University of Belgrade, 11000 Belgrade, Serbia
^2^ Somogy Megyei Kaposi Mór Oktató Kórház, 7400, Hungary
^3^ Clinic of Hematology, University Hospital St. Marina, 9010 Varna, Bulgaria
^4^ Institute of Hematology and Transfusion Medicine, Department of Haematology, 00-791 Warsaw, Poland
^5^ Institute of Pathology, University Clinic Wuppertal, University of Witten / Herdecke, Germany
^6^ Research & Development, Shire International GmbH (a member of the Takeda group of companies), 6300 Zug, Switzerland
^7^ Research & Development, Shire (a member of the Takeda group of companies), Lexington, MA 02421, USA

**Correspondence to:** Professor Mirjana Gotic, Clinic for Hematology Clinical Center of Serbia, University of Belgrade School of Medicine, Koste Todorovica 2, Belgrade 11000, Serbia.
**E-mail:** [miragotic@yahoo.com](mailto:miragotic@yahoo.com)

*Additional inclusion and exclusion criteria*

Additional inclusion criteria included: lack of Philadelphia chromosome or *bcr/abl* gene rearrangement, absence of collagen fibrosis of marrow within 6 months prior to screening or restricted to <1/3 of biopsy area without marked splenomegaly and leucoerythroblastic peripheral blood changes, no evidence of myelodysplastic syndrome, no cause for reactive thrombocytosis, and satisfactory medical assessment with no clinically significant or relevant abnormalities.

Additional exclusion criteria included: prior or current treatment with cytoreductive therapy; use of anticoagulants; use of any therapy known to affect ventricular ejection fraction (i.e., positive inotropes, diuretics, antiarrhythmic drugs, beta blockers, antihypertensives, drugs affecting the renin-angiotensin system, nitrates, calcium channel blockers, potassium channel activators, and sympathomimetics); or use of anti-aggregant therapies, including aspirin (aspirin and other anti-aggregant therapies were permitted until randomisation). Use of aspirin was excluded to avoid any interactions between aspirin and anagrelide and/or hydroxyurea. Female patients who were pregnant, lactating or of childbearing age and not using reliable methods of contraception were also excluded.

**Online resource 1A.** Mean platelet count and mean change from baseline at 3 and 36 months in full analysis set population (FAS, patients who received ≥1 dose of study medication and had a pre-treatment and ≥1 post-baseline LVEF measurement)

| **Study visit** | **Anagrelide  N = 73** | **Hydroxyurea  N = 68** | |  |
| --- | --- | --- | --- | --- |
| Month 3 |  | |  |  |
| LS mean (SE)^a^ | 575.3 (36.11) | | 462.2 (37.57) |  |
| LS mean difference (95% CI)^b,c^ |  | |  | –113.1 (–187.40, –38.83) |
| Month 36 |  | |  |  |
| LS mean (SE)^a^ | 531.0 (42.14) | | 462.8 (43.81) |  |
| LS mean difference (95% CI)^b,c^ |  | |  | –68.3 (–154.95, 18.43) |

ANCOVA, analysis of covariance; CI, confidence interval; LOCF, last observation carried forward; LS, least squares; SE, standard error.

Baseline corresponds to the first valid (non-missing interpretation) observation obtained at the baseline visit. If the baseline was missing, the screening value was used.

^a^If value was missing at the visit, LOCF imputation was used, considering all post-baseline data prior to the missing time point; ^b^From ANCOVA model including treatment, age category, presence of previous thrombosis/haemorrhage as main effects and baseline platelet count as a covariate; ^c^Noninferiority of anagrelide could be concluded if lower limit of 95% CI for the difference between treatment groups (hydroxyurea minus anagrelide) was > -100 x 10^9^/L.

**Online Resource 1b** Mean platelet count and mean change from baseline at 3 and 36 months in per-protocol population (PP population, FAS patients with no major protocol deviations)

| **Study visit** | **Anagrelide  N = 22** | **Hydroxyurea  N = 38** |  |
| --- | --- | --- | --- |
| Month 3 |  |  |  |
| LS mean (SE)^a^ | 460.7 (47.41) | 375.1 (45.75) |  |
| LS mean difference (95% CI)^b,c^ |  |  | –85.6 (–179.12, 7.85) |
| Month 36 |  |  |  |
| LS mean (SE)^a^ | 362.4 (54.37) | 429.4 (52.47) |  |
| LS mean difference (95% CI)^b,c^ |  |  | 67.0 (–40.17, 174.26) |

ANCOVA, analysis of covariance; CI, confidence interval; LOCF, last observation carried forward; LS, least squares; SE, standard error.

Baseline corresponds to the first valid (non-missing interpretation) observation obtained at the baseline visit. If the baseline was missing, the screening value was used.

^a^If value was missing at the visit, LOCF imputation was used, considering all post-baseline data prior to the missing time point; ^b^From ANCOVA model including treatment, age category, presence of previous thrombosis/haemorrhage as main effects and baseline platelet count as a covariate; ^c^Noninferiority of anagrelide could be concluded if lower limit of 95% CI for the difference between treatment groups (hydroxyurea minus anagrelide) was > -100 x 10^9^/L.

**Differences between the FAS and PP populations**

Most patients had ≥1 major protocol deviation (51 subjects (61.9%) in the anagrelide group vs. 30 subjects (44.1%) in the hydroxyurea groups). These deviations were due to the use of disallowed medication (25 subjects [34.2%] in the anagrelide treatment group and 15 subjects [22.1%] in the HU treatment group), or lack of centrally confirmed ET diagnosis. . Multiple subjects using disallowed medication continued in the study and were not detected as protocol deviators until a later date.

The PP population excluded all patients that had a major protocol deviation, whilst the FAS population included all patients.

**Online resource 1c** *Post-hoc* analysis of mean platelet count and mean change from baseline at 3 and 36 months in the full analysis set population

|  | **Mixed-effects model** | | | | | **Observed data** | | | | |  |
| --- | --- | --- | --- | --- | --- | --- | --- | --- | --- | --- | --- |
| **Study visit** | **Anagrelide  N = 73** | | **Hydroxyurea  N = 68** | |  | | **Anagrelide  N = 73** | | **Hydroxyurea  N = 68** | |  |
| Month 3 |  |  | |  | |  | |  | |  |  |
| n | 67 | 62 | |  | | 72 | | 66 | |  |  |
| LS mean (SE)^a^ | 512.1 (35.23) | 413.1 (35.88) | |  | | 504.5 (27.27) | | 401.8 (28.23) | |  |  |
| LS mean difference (95% CI)^b,c^ |  |  | | –99.0  (–166.45, –31.55) | |  | |  | | –102.7  (–169.63, –35.83) |  |
| Month 36 |  |  | |  | |  | |  | |  |  |
| n | 40 | 43 | |  | | 72 | | 66 | |  |  |
| LS mean (SE)^a^ | 389.2 (27.81) | 447.1 (28.56) | |  | | 415.4 (25.60) | | 453.3 (25.50) | |  |  |
| LS mean difference (95% CI)^b,c^ |  |  | | 57.9 (1.58, 114.24) | |  | |  | | 37.9  (–22.23, 97.96) |  |

ANCOVA, analysis of covariance; CI, confidence interval; LOCF, last observation carried forward; LS, least squares; SE, standard error.

Baseline corresponds to the first valid (non-missing interpretation) observation obtained at the baseline visit. If the baseline was missing, the screening value was used.

^a^If value was missing at the visit, LOCF imputation was used, considering all post-baseline data prior to the missing time point; ^b^From ANCOVA model including treatment, age category, presence of previous thrombosis/haemorrhage as main effects and baseline platelet count as a covariate; ^c^Noninferiority of anagrelide could be concluded if lower limit of 95% CI for the difference between treatment groups (hydroxyurea – anagrelide) was >-100 x 10^9^/L.
